# Evaluation of accuracy, quality, and readability of information on hypothyroidism provided by different artificial intelligence chatbot models

**DOI:** 10.3389/fpubh.2025.1698596

**Published:** 2025-12-10

**Authors:** Ting Ruan, Xinran Shao, Yihan Sun, Xingai Ju, Jianchun Cui

**Affiliations:** 1 Liaoning University of Traditional Chinese Medicine, Shenyang, China; 2 Department of Thyroid and Breast Surgery, China Medical University People's Hospital, Shenyang, China; 3 Department of Cardiology, China Medical University People's Hospital, Shenyang, China; 4 Department of General Medicine, China Medical University People's Hospital, Shenyang, China

**Keywords:** artificial intelligence chatbot, hypothyroidism, readability, clinical guideline, patient education

## Abstract

**Objective:**

This study assessed the accuracy, quality, and readability of responses from three leading AI chatbots—ChatGPT-3.5, DeepSeek-V3, and Google Gemini-2.5—on the diagnosis, treatment, and long-term risks of adult hypothyroidism, comparing their outputs with current clinical guidelines.

**Methods:**

Two thyroid specialists developed 27 questions based on the Guideline for the Diagnosis and Management of Hypothyroidism in Adults (2017 edition), covering three categories: diagnosis, treatment, and long-term health risks. Responses from each AI model were independently evaluated by two reviewers. Accuracy was rated using a six-point Likert scale, quality using the DISCERN tool and the five-point Likert scale, and readability was assessed by the Flesch Reading Ease (FRE), Flesch–Kincaid Grade Level (FKGL), Gunning Fog Index (GFI),and Simple Measure of Gobbledygook(SMOG).

**Results:**

All three AI models demonstrated excellent performance in accuracy (mean score > 4.5) and quality (high-quality rate > 94%). According to the DISCERN tool, no significant difference was observed in the overall information quality among the models. However, Gemini-2.5 generated responses of significantly lower quality for treatment-related questions than for diagnostic inquiries. The content generated by all models was relatively difficult to comprehend (low FRE scores and high FKGL/GFI scores), generally requiring a college-level or higher education for adequate understanding.

**Conclusion:**

All three AI chatbots were capable of producing highly accurate and high-quality medical information regarding hypothyroidism, with their responses showing strong consistency with clinical guidelines. This underscores the substantial potential of AI in supporting medical information delivery. However, the consistently high reading difficulty of their outputs may limit their practical utility in patient education. Future research should focus on improving the readability and patient-friendliness of AI outputs—through prompt engineering and multi-round dialogue optimization—while maintaining professional accuracy, to enable broader application of AI in health education.

## 1 Introduction

Hypothyroidism has a relatively high global prevalence, with the NHANES III study reporting an overall rate of 4.6% (1). Its clinical presentation is often nonspecific and insidious in onset, with severity varying according to the degree of thyroid dysfunction. Common symptoms include fatigue, cold intolerance, dry skin, constipation, voice changes, and muscle aches. Severe hypothyroidism can even negatively affect both maternal and fetal health during pregnancy (2). The broad spectrum of symptoms highlights its profound impact on metabolism and multiple organ systems, underscoring the importance of early diagnosis, standardized treatment, and systematic follow-up for effective disease management. Patients often experience anxiety about their condition while struggling to access accurate information, leading many to turn to the internet as their primary source of health-related knowledge (3). However, the variable quality and sheer volume of health information available online pose a significant challenge for laypersons in assessing its credibility and validity, thereby underscoring the critical need for reliable and comprehensible health communication. This context establishes hypothyroidism as an ideal case study for evaluating AI chatbots as patient education tools. The quality and readability of AI-generated responses for such chronic conditions—where long-term management and patient education are paramount—differ fundamentally from their utility in acute or diagnostic scenarios, primarily due to vast differences in patient information needs and health literacy levels. Reliable information on hypothyroidism should encompass both the short- and long-term consequences of the disease. This is essential for helping patients fully understand their condition, alleviate anxiety, and take proactive measures to manage and mitigate the lifelong impact of hypothyroidism.

Artificial intelligence (AI) chatbots are virtual assistants powered by natural language processing technologies and deployed via social media or online platforms. In recent years, AI chatbots have seen rapid growth in healthcare applications (4). For example, ChatGPT has demonstrated excellent performance in addressing open-ended medical questions and multiple-choice examinations (5). Gemini has shown outstanding accuracy in ophthalmology-related queries, achieving a professional standard comparable to that of medical residents (6). DeepSeek has been able to provide well-structured and comprehensive responses on common otolaryngological procedures, including indications, treatment options, and surgical risks (7). With advantages such as ease of use and rapid response, AI chatbots can provide real-time decision support for patients seeking information and for healthcare professionals in need of quick, reliable insights. Both patients and healthcare providers may increasingly shift from traditional internet searches to dynamic, interactive, AI-driven modes of real-time information acquisition (8). This trend underscores both the promise and challenges of managing hypothyroidism through such platforms, as individuals with limited medical knowledge may struggle to evaluate the reliability and validity of the information they receive. Therefore, ensuring the accuracy of AI-generated information and decision-making in such critical healthcare contexts is of paramount importance.

Previous research has predominantly focused on evaluating the factual accuracy and informational quality of AI-generated chatbot responses, while largely overlooking the equally critical dimension of readability. Although AI chatbots can produce technically accurate medical information, their practical utility as patient education tools is significantly compromised if the content remains incomprehensible to patients with varying levels of health literacy and education. Furthermore, although multi-model evaluations exist in other fields and initial investigations have begun to explore AI applications in thyroid diseases (9), research systematically integrating accuracy, quality, and patient readability specifically within the context of hypothyroidism remains scarce. A significant gap persists in the systematic, multidimensional comparative evaluation of multiple leading AI models for this particular chronic condition, where balancing professional authority with information accessibility is crucial. To address this gap, this study systematically assessed the accuracy, quality, and readability of three widely used AI chatbots—ChatGPT-3.5, DeepSeek-V3, and Gemini-2.5—by comparing their responses to hypothyroidism-related questions against clinical guidelines. This study innovatively incorporates readability and patient-friendliness as key assessment dimensions alongside accuracy to evaluate AI-generated content on hypothyroidism, a condition requiring long-term patient education. It thus critically examines AI’s role not just for information accuracy but as a practical tool for sustained patient education.

## 2 Materials and methods

### 2.1 Study design

This was a cross-sectional study involving three AI chatbot models—ChatGPT-3.5^1^, DeepSeek-V3^2^, and Gemini-2.5^3^—to evaluate their performance in answering questions related to adult hypothyroidism and to assess their potential for medical applications. We utilized the public web interface with models operating under default, platform-optimized settings that balance response creativity with factual accuracy. While this approach differs from strictly controlled API access, it maximizes the ecological validity of our findings by simulating typical public usage scenarios in real-world contexts. The questions were developed by two senior thyroid specialists based on the most recent *Guideline for the Diagnosis and Management of Hypothyroidism in Adults (2017 edition)* (10). A total of 27 questions were included (Table 1), categorized into three domains: diagnosis (5 questions), long-term health risks (4 questions), and treatment strategies (18 questions). As this study did not involve human or animal experimentation, no ethical approval or informed consent was required.

**Table 1:** The questions posed to AI models.

Classification	Questions
Prompt	Imagine you are an experienced Thyroid surgeon with a knowledgeable background in the latest research in the field of Thyroid. Answer the following prompts based on evidence-based research studies and point out any lack of evidence to support a point (point out if there is a lack of study to support or refute your answer).
Diagnosis	Q1: What are the diagnostic indicators of primary hypothyroidism?
	Q2: What is the diagnostic basis for hypothyroidism and subclinical hypothyroidism during pregnancy?
	Q3: What is the basis for diagnosis of subclinical hypothyroidism?
	Q4: What are the classification and criteria for subclinical hypothyroidism?
	Q5: What’s the Differential diagnosis of thyroid hormone resistance syndrome (RTH) and hypothyroidism?
Long-term health risks	Q1: What are the risk factors associated with mild subclinical hypothyroidism? It is recommended to take treatment and what kind of treatment?
	Q2: What are the risks of clinical hypothyroidism during pregnancy? Does it need treatment?
	Q3: When women of childbearing age who have suffered from hypothyroidism or subclinical hypothyroidism plan to be pregnant, they need to adjust and monitor the dynamotherapeutic indicators to what extent can they be pregnant?
	Q4: The long-term harm of hypothyroidism and its concomitant critical and severe diseases and their treatment strategies?
Treatment plan	Q1: What are the treatment goals of primary hypothyroidism?
	Q2: What are the alternative drug treatments for hypothyroidism? Q3: How to formulate the dosage of hypothyroidism replacement therapy?
	Q4: How to determine the initial dose of hypothyroid replacement therapy and the time it takes to achieve a complete replacement dose?
	Q5: What is the medication for L-T4?
	Q6: What are the suggestions for the use of L-T3 in hypothyroidism treatment?
	Q7: What are the suggestions for the use of dry thyroid tablets in the treatment of hypothyroidism?
	Q8: What are the suggestions for combination medications in hypothyroidism treatment?
	Q9: What indicators need to be monitored during which time periods to achieve treatment goals when supplementing L-T4 treatment?
	Q10: Treatment strategies and treatment goals for severe subclinical hypothyroidism?
	Q11: What are the drug treatment options for hypothyroidism and subclinical hypothyroidism during pregnancy?
	Q12: What is the clinical hypothyroidism treatment plan for pregnancy diagnosis?
	Q13: What are the serological goals of hypothyroidism and subclinical hypothyroidism treatment during pregnancy?
	Q14: What is the monitoring method for hypothyroidism-related serological indicators during pregnancy?
	Q15: Treatment recommendations for women with subclinical hypothyroidism during pregnancy?
	Q16: How to monitor serological indicators after childbirth and formulate a time for discontinuation based on this?
	Q17: What are the targets for hypothyroidism secondary to the hypothalamus and pituitary gland?
	Q18: What are the treatment suggestions for pathological syndromes with normal thyroid function?

### 2.2 Analysis of AI model responses

Two thyroid specialists independently posed the questions to ChatGPT-3.5, DeepSeek-V3, and Gemini-2.5. Each query was accompanied by a standardized prompt. For example, imagine you are an experienced Thyroid surgeon with a knowledgeable background in the latest research in the field of Thyroid. Answer the following prompts based on evidence-based research studies and point out any lack of evidence to support a point (point out if there is a lack of study to support or refute your answer). What are the diagnostic indicators of primary hypothyroidism? The prompt strategy in this study was designed to simulate real-world public interactions with AI. To enhance the reproducibility and structural integrity of prompt engineering, we adopted the emotion-aware embedding fusion framework proposed by Rasool et al. (11). This systematic prompt design incorporates multi-level emotional lexicons and attention mechanisms, ensuring accuracy and consistency of AI responses within professional contexts.

**Figure 1:**
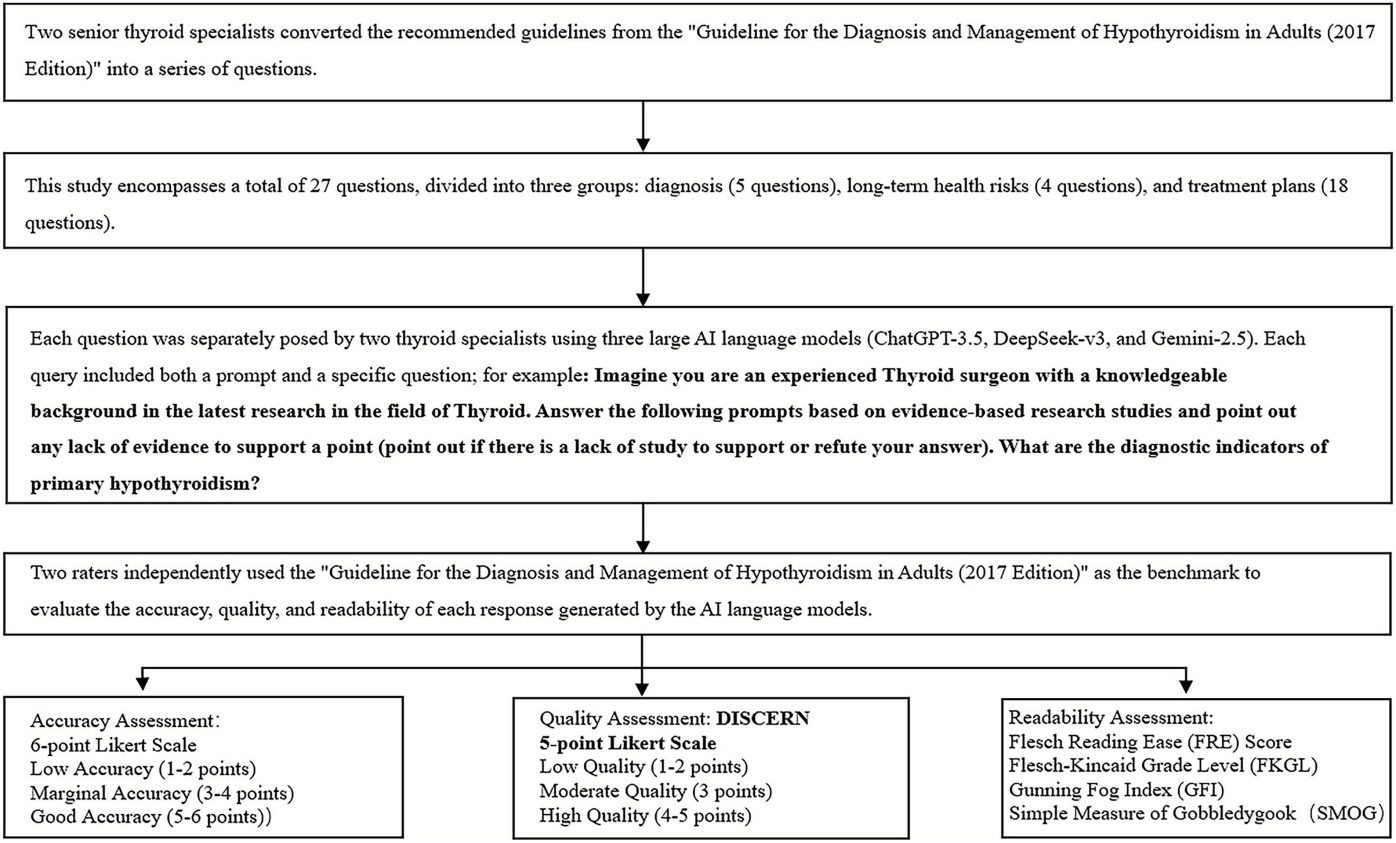
Research cohort diagram.

To ensure objectivity, evaluators are blinded to the source AI model of each response. Reviewers independently assessed the accuracy, quality, and readability of the responses against the 2017 clinical guideline. Accuracy is rated on a six-point Likert scale ranging from 1 (completely incorrect) to 6 (completely correct), and classified into three categories: low accuracy (1, 2), borderline accuracy (3, 4), and good accuracy (5, 6). Quality was assessed using the DISCERN instrument and a 5-point Likert scale. The DISCERN instrument is a validated tool designed to evaluate the quality and reliability of written healthcare information, particularly concerning treatment choices. It comprises 16 items, each scored on a scale of 1 to 5. These items are categorized into three sections: assessing information reliability, specific treatment options, and overall quality. The total score was used to grade the overall reliability of the text. As the original DISCERN publication does not specify a grading cutoff, the criteria established in prior literature were adopted for this study (12). The 5-point Likert scale ranged from 1 (poor quality) to 5 (excellent quality), and was grouped into three levels: low (1, 2), moderate (3), and high quality (4, 5). Readability is evaluated using four widely recognized indices: the Flesch Reading Ease (FRE) score, the Flesch–Kincaid Grade Level (FKGL), the Gunning Fog Index (GFI), and the Simple Measure of Gobbledygook (SMOG). The research cohort diagram is shown in Figure 1.

### 2.3 Data analysis

The Kolmogorov–Smirnov test was used to assess the normality of data distribution. Continuous variables were expressed as mean ± standard deviation, while categorical variables were presented as percentages. Accuracy and quality scores of each model were reported as frequencies and percentages; intergroup comparisons were performed using the chi-square test. One-way ANOVA was conducted to compare the three models across accuracy, quality, and readability metrics. Statistical analyses were performed using SPSS version 26.0, with a *p* value < 0.05 considered statistically significant.

## 3 Results

### 3.1 Accuracy assessment

Inter-rater reliability was assessed using the Pearson correlation coefficient, ranging from −1 to 1, with higher values indicating stronger agreement (>0.7 = strong correlation, 0.3–0.7 = moderate, <0.3 = weak). The inter-rater correlation coefficients were 0.68 for ChatGPT-3.5 (moderate agreement), 0.72 for DeepSeek-V3 (strong agreement), and 0.65 for Gemini-2.5 (moderate agreement). The accuracy ratings between the two reviewers showed at least moderate agreement, indicating reliability and allowing for subsequent comparative analysis.

**Figure 2:**
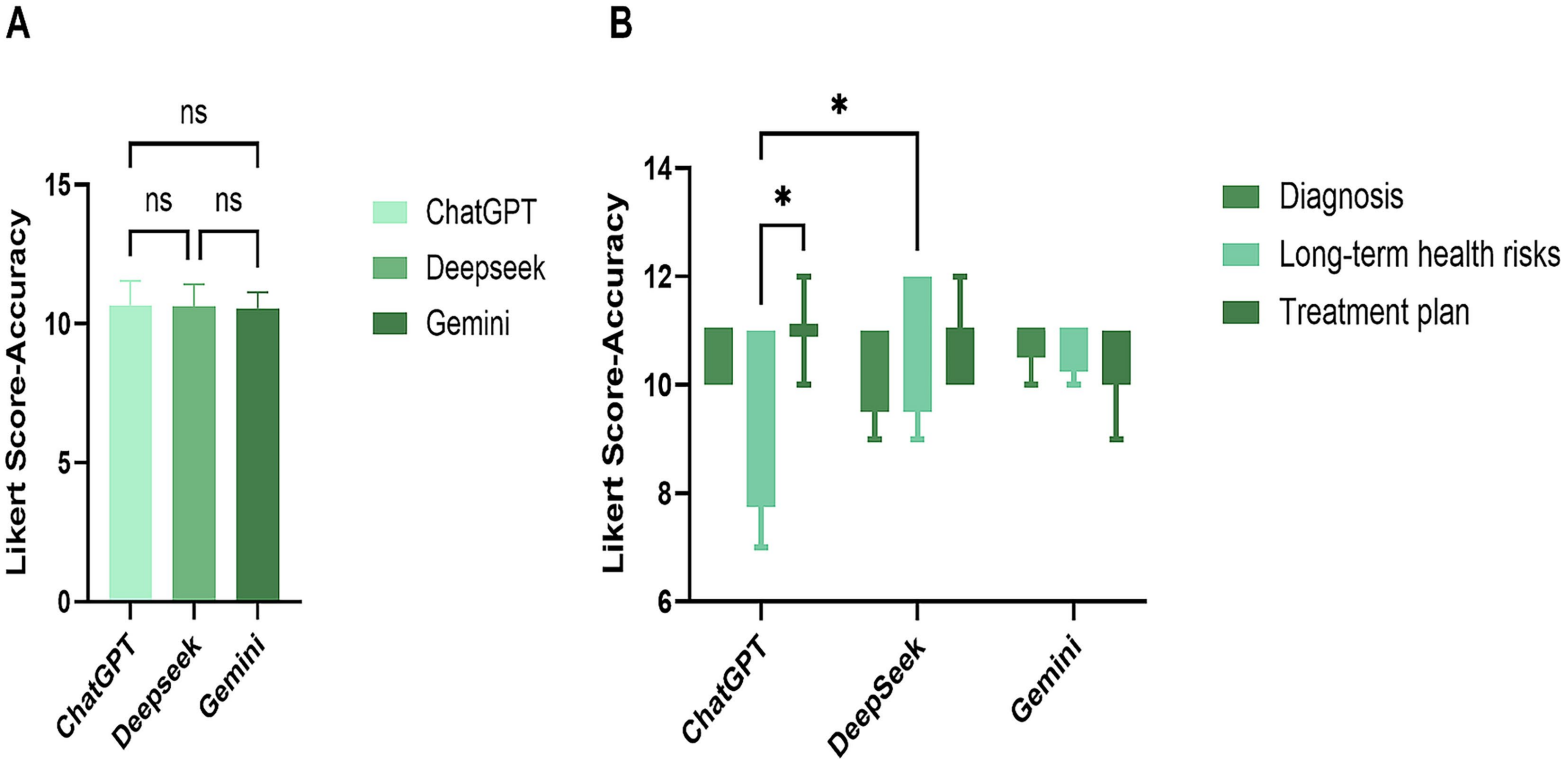
Overall and intergroup accuracy analysis. The horizontal coordinate is the name of three language models, the vertical coordinates and numerical values are the scores of Likert scales. Panel **(A)** is the overall accuracy analysis result of the three models, and panel **(B)** is the accuracy analysis result of each model on different categories of problems.

The accuracy scores of all three AI models followed a normal distribution and demonstrated homogeneity of variance. Scores for all three models clustered in the high range (5–6 points, >85% of responses), with no extreme low scores (1–2 points), indicating overall good accuracy. Based on mean accuracy scores, the ranking was: ChatGPT-3.5 (5.56) > Gemini-2.5 (5.41) > DeepSeek-V3 (5.30). Further analyses of accuracy by question category are shown in Figure 2. No significant intergroup differences were observed in overall accuracy across the 27 guideline-based questions, though some differences emerged within specific categories. For diagnostic and treatment-related questions, no significant differences in accuracy were noted among the three models. In responses to long-term risk questions, DeepSeek-V3 showed slightly higher accuracy compared to ChatGPT-3.5. Within-model analysis revealed that ChatGPT-3.5 performed better on treatment-related questions than on long-term risk questions. Overall, the greatest variability in accuracy was observed in long-term risk questions, where DeepSeek-V3 performed best, while ChatGPT-3.5 showed stronger performance in diagnostic and treatment-related queries. Importantly, despite some intergroup differences, all models achieved mean scores above 4.5 across categories, with no low scores (1–3 points), indicating consistently good accuracy across models.

### 3.2 Quality assessment

Quality was evaluated using the DISCERN instrument and a 5-point Likert scale. This approach encompassed both subjective quality ratings and quantifiable metrics of reliability and presentation quality. Responses from the three AI models were independently evaluated by two thyroid specialists. Given the subjective nature of the assessment metrics, inter-rater reliability (IRR) was calculated to verify the consistency of the scoring. Pearson correlation coefficients were used to analyze IRR for the quality ratings across all 27 questions. The IRR values for the three AI models ranged between 0.53 and 0.55: ChatGPT-3.5 (*r* = 0.54), DeepSeek-V3 (*r* = 0.53), and Gemini-2.5 (*r* = 0.55). This indicates a moderate level of agreement between the two specialists in their judgment of response quality. Although quality assessment involves subjective evaluation—encompassing comprehensive judgments of information completeness, structural coherence, and reliability—this consistency demonstrates that the two independent specialists shared similar understandings and criteria for defining “high-quality” and “low-quality” responses. The obtained reliability data thus provides a solid foundation for the analysis of quality differences among the models presented in the Results section.

The DISCERN scores, ranging from 15 to 75, were categorized into five quality levels: very low (13–24), low (27–38), moderate (39–50), good (51–62), and excellent (63–75). Higher DISCERN scores indicate a higher quality of health information. The scores for the three AI chatbot models are summarized in Table 2. The mean scores of ChatGPT-3.5 (56.74) and Gemini-2.5 (56.41) were comparable and slightly higher than that of DeepSeek-V3 (54.59). Gemini-2.5 exhibited the largest score variability, with the highest single score (65), the lowest (45.5), and the greatest standard deviation (± 6.22), indicating less consistent response quality compared to the other models. Overall, no statistically significant difference was observed in the total DISCERN scores among the three AI models.

**Table 2:** Analysis of DISCERN scores among AI Chatbot models.

AI model	Diagnosis	Long-term health risks	Treatment	Overall	*p*-value (Intra-AI Comparison)^1^
ChatGPT-3.5	Max: 55.5	Max: 62.5	Max: 63.0	Max: 65.0	0.4497
	Min: 47.0	Min: 54.0	Min: 50.0	Min: 46.0	
	Mean ± SD: 52.10 ± 3.58	Mean ± SD: 58.63 ± 4.56	Mean ± SD: 58.00 ± 4.61	Mean ± SD: 56.74 ± 5.06	
	Rating: Good	Rating: Good	Rating: Good	Rating: Good	
DeepSeek-V3	Max: 56.5	Max: 59.5	Max: 61.5	Max: 62.0	0.0583
	Min: 52.0	Min: 53.5	Min: 47.0	Min: 47.0	
	Mean ± SD: 54.10 ± 1.97	Mean ± SD: 57.25 ± 2.49	Mean ± SD: 53.00 ± 4.14	Mean ± SD: 54.59 ± 4.09	
	Rating: Good	Rating: Good	Rating: Good	Rating: Good	
Gemini-2.5	Max: 63.5	Max: 64.0	Max: 64.5	Max: 65.0	0.0117*
	Min: 59.0	Min: 53.0	Min: 45.0	Min: 45.5	**P**^**1**^ **= 0.0102**
	Mean ± SD: 61.10 ± 2.11	Mean ± SD: 58.75 ± 5.06	Mean ± SD: 50.89 ± 6.23	Mean ± SD: 56.41 ± 6.22	P^2^ = 0.0652
	Rating: Good	Rating: Good	Rating: Fair	Rating: Good	P^3^ = 0.8118
*p*-value (Inter-AI Comparison)^2^	0.0016*	0.8127	0.0152*	0.4447	

*p*-values < 0.05 are considered statistically significant and are marked with bold and asterisks *.

^1^Intra-AI Comparison (Row): Compares the scores of a single AI across the ‘Diagnosis’, ‘Long-term Health’, and ‘Treatment’ categories (Kruskal-Wallis test).

^2^Inter-AI comparison (Column): compares the scores among the three AIs for each category and for the overall score (Kruskal-Wallis test).

^3^Dunn’s *post hoc* test *p*-value matrix: P^1^: diagnosis vs treatment, P^2^: long-term health vs treatment, P^3^: diagnosis vs long-term health.

However, the response quality of Gemini-2.5 differed significantly across question categories. Post-hoc analysis revealed that this disparity was primarily driven by the significantly lower quality of its responses to “treatment”-related questions compared to “diagnostic” questions. Specifically, while Gemini-2.5 provided high-quality responses for diagnostic and long-term health issues (median score ~60), its performance markedly declined for treatment-related questions (median score 50.5). A direct comparison of models within each category showed that Gemini-2.5 excelled in diagnostic questions but was weakest on treatment-related topics. In contrast, ChatGPT-3.5 and DeepSeek-V3 provided more reliable responses for treatment-related questions. Among the three models, ChatGPT-3.5 demonstrated the most consistent performance across all question types, whereas Gemini-2.5’s performance was highly variable. ChatGPT-3.5 demonstrated the most stable performance across all questions, whereas Gemini-2.5 showed high variability dependent on question type.

The 5-point Likert scale ranged from 1 (poor) to 5 (excellent) and was categorized into three levels: low (1–2), moderate (3), and high (4–5) quality. Frequency analysis of quality ratings (Table 3) revealed a low incidence of low-quality scores. Notably, no low-quality ratings were assigned to either DeepSeek-V3 or Gemini-2.5. The overall high-quality response rate exceeded 94% for all models, indicating consistently high output quality.

**Table 3:** Frequency statistics of quality scores.

Model	Low quality (1–2 points)	Medium quality (3 points)	High quality (4–5 points)	Mean ± SD
ChatGPT	1(1.9%)	2 (3.7%)	51 (94.4%)	4.56 ± 0.57
DeepSeek	0 (0.0%)	1(1.9%)	53 (98.1%)	4.56 ± 0.53
Gemini	0 (0.0%)	3 (5.6%)	51 (94.4%)	4.63 ± 0.59

*χ*^2^ (4) = 4.74, *p* = 0.315.

**Figure 3:**
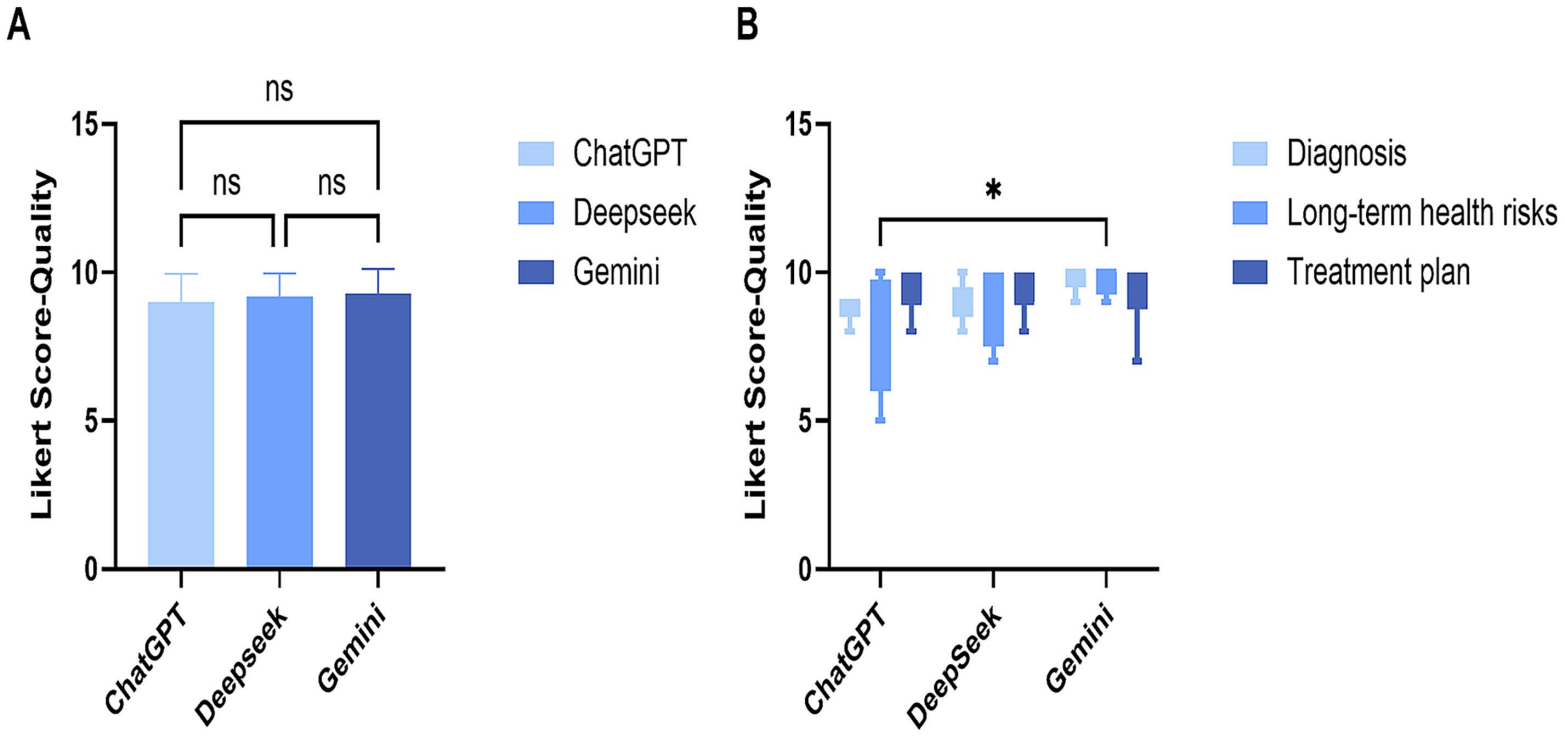
Overall and intergroup quality analysis. The horizontal coordinate is the name of three language models, the vertical coordinates and numerical values are the scores of Likert scales. Panel **(A)** is the overall quality analysis result of the three models, and Panel **(B)** is the quality analysis result of each model on different categories of problems.

Figure 3 further illustrates the quality score differences among models and across the three categories of questions. Results showed no statistically significant differences in response quality among ChatGPT-3.5, DeepSeek-V3, and Gemini-2.5. The distribution of low, moderate, and high-quality scores was similar across models, with all receiving consistently high ratings. In subgroup analysis, ChatGPT-3.5 and Gemini-2.5 differed in quality scores for long-term risk questions, with ChatGPT-3.5 showing less stability and greater score variability compared to the other models. In contrast, DeepSeek-V3 and Gemini-2.5 demonstrated more stable quality performance across question categories.

### 3.3 Readability assessment

Readability of chatbot responses was assessed using the Flesch Reading Ease (FRE) score, the Flesch–Kincaid Grade Level (FKGL), the Gunning Fog Index (GFI), and the Simple Measure of Gobbledygook (SMOG). These metrics are derived through predefined mathematical formulas that account for textual features such as sentence length, syllable count, and the number of polysyllabic words. As readability indices represent purely objective, automated computational metrics, their values are entirely determined by the input text. Provided the input text remains unchanged, recalculating these indices using any standardized online tool or software package will yield perfectly consistent results. Consequently, there is no subjective variability between different “raters” of these computational metrics, and their theoretical inter-rater reliability reaches 1.00 (perfect agreement). Therefore, conducting an inter-rater reliability (IRR) analysis for such automated indices is methodologically unnecessary.

FRE scores, ranging from 0 to 100, are calculated based on syllable count and sentence length, with higher values indicating greater ease of reading (25). The FKGL translates readability into corresponding U.S. grade levels (0–20), estimating the minimum educational attainment required for comprehension; lower scores indicate easier readability (26). The GFI evaluates vocabulary complexity (e.g., long or multisyllabic words) and its impact on reading comprehension, with scores from 6 to 20 corresponding to U.S. grade levels; higher scores indicate a higher level of education required. The Simple Measure of Gobbledygook (SMOG) index estimates text readability based on syntactic complexity, which calculates a grade level by factoring in the number of polysyllabic words (three or more syllables) per sentence (13). Readability scores were calculated using an online tool.^4^ The classification ranges for the four indices are shown in Table 4.

**Table 4:** The score ranges and classification levels for readability indices.

FRE		FKGL		GFI		SMOG	
Score range	Level	Score range	Level	Score range	Level	Score range	Level
90–100	Very easy	<5.0	Elementary (Lower)	<6.0	Elementary (Middle)	3–6	3rd–6th Grade/Easy
80–89	Easy	5.0–6.9	Elementary (Upper)	6.0–8.9	Elementary/Junior high	7–8	7th–8th Grade/Average
70–79	Fairly easy	7.0–9.9	Junior High	9.0–10.9	High School (Lower)	9–12	9th–12th Grade/Difficult
60–69	Standard	10.0–12.9	High School	11.0–12.9	High School Graduate	13–16	College (1st–4th Year)/Very Difficult
50–59	Fairly difficult	13.0–16.9	College/University	13.0–16.9	University	17+	Graduate Level and above/Complex
30–49	Difficult	≥17.0	Graduate/Professional	≥17.0	Professional/Expert		
0–29	Very difficult						

A single readability metric may not fully capture the impact of conceptual complexity or domain-specific terminology; relying solely on one indicator is insufficient for a comprehensive evaluation of text readability. For instance, certain texts may contain short sentences and simple wording (yielding a higher FRE score) while still addressing highly specialized concepts. In contrast, the GFI and FKGL emphasize the educational grade level required to comprehend the text, thereby providing a more direct link to the reader’s educational background. The combined use of all four indices thus enables a multidimensional assessment of readability, enhancing the validity of the evaluation. To compare overall differences in readability across the three models, one-way ANOVA was conducted separately for FRE, FKGL, GFI, and SMOG scores. When significant differences were identified (*p* < 0.05), *post hoc* tests were performed to specify inter-model and inter-category variations. The statistical results of readability scores for the three models are presented in Table 5.

**Table 5:** Chaobot model readability score statistics.

Category	Indicators	ChatGPT		Deepseek		Gemini	
		Mean ± SD	(Min/Max)	Mean ± SD	(Min/Max)	Mean ± SD	(Min/Max)
Overall	FRE	21.67 ± 8.16ᵇ	(8/35)	32.59 ± 14.96ᵃ	(0/51)	18.70 ± 20.94ᵃ	(0/64)
	GFI	15.01 ± 1.13ᵃ	(13.3/18)	14.56 ± 2.09ᵇ	(12/20.2)	18.23 ± 3.23ᵇ	(11.9/23.8)
	FKGL	14.77 ± 1.39ᵃ	(12.76/18.28)	11.58 ± 2.92ᵇ	(8.24/19.37)	19.14 ± 4.67ᵇ	(9.28/28.34)
	SMOG	12.44 ± 3.19^a^	(2.08/15.10)	10.23 ± 2.21^b^	(7.82/16.29)	15.86 ± 3.52^c^	(10.46/22.05)
Diagnosis	FRE	18.20 ± 6.42ᶜ	(13/28)	31.40 ± 12.74ᵇ	(13/43)	0.40 ± 0.89ᵃ	(0/2)
	GFI	15.04 ± 0.90ᵃ	(14.1/16.2)	14.40 ± 1.10ᵃ	(13.4/15.8)	21.62 ± 1.58ᵇ	(20/23.8)
	FKGL	13.96 ± 1.49ᵃ	(12.76/16.11)	11.59 ± 1.63ᵃ	(9.26/12.78)	24.14 ± 3.19ᵇ	(20.56/28.34)
	SMOG	9.28 ± 4.41^a^	(2.08/13.92)	8.96 ± 1.39^b^	(7.82/11.08)	19.64 ± 1.88^c^	(17.38/22.05)
Long-term health risks	FRE	14.25 ± 5.85ᵇ	(8/21)	17.5 ± 12.37ᵃᵇ	(0/30)	3.50 ± 7.00ᵃ	(0/14)
	GFI	16.73 ± 1.05ᵃ	(15.6/18)	15.09 ± 2.66ᵃᵇ	(15.1/20.2)	20.08 ± 1.94ᵇ	(17.5/22.1)
	FKGL	15.19 ± 1.14 ᵃ	(14.15/16.77)	13.57 ± 2.19 ᵃᵇ	(12.83/19.37)	22.28 ± 3.99 ᵇ	(18.52/27.02)
	SMOG	12.54 ± 0.88^a^	(12.00/13.88)	13.23 ± 2.05^b^	(11.86/16.29)	18.06 ± 3.03^c^	(14.96/21.45)
Treatment plan	FRE	23.50 ± 7.15ᵇ	(13/35)	36.44 ± 12.87ᵃ	(8/51)	26.61 ± 20.66ᵇ	(0/64)
	GFI	14.80 ± 0.95ᵃ	(13.3/16.8)	14.08 ± 1.70ᶜ	(12/17.6)	17.47 ± 2.92ᵇ	(11.9/22.5)
	FKGL	14.96 ± 1.34ᵃ	(13.03/18.28)	10.96 ± 2.22ᶜ	(8.24/16.29)	18.27 ± 4.40ᵇ	(9.7/26.11)
	SMOG	12.77 ± 0.98^a^	(11.18/14.47)	9.92 ± 2.05^b^	(8.14/15.13)	14.23 ± 2.12^c^	(10.46/16.24)

Cell values: All data in the table are presented as mean ± standard deviation. A higher FRE score indicates greater readability, whereas lower GFI and FKGL scores denote easier readability. Superscript letters (a, b, c): Within the same row, means with different superscript letters differ significantly (*p* < 0.05), whereas means sharing the same letter are not significantly different.

Collectively, the FRE, FKGL, and SMOG indices revealed highly significant differences (*p* < 0.05) among the three AI models, indicating systematic variations in the readability of their generated texts. Although the GFI showed no significant difference between DeepSeek-V3 and Gemini-2.5 (*p* = 0.082), suggesting similarities in their handling of complex vocabulary and syntax, significant differences were observed when each was compared to ChatGPT-3.5. Despite these significant inter-model differences reflecting distinct content generation capabilities, the outputs from all three models were universally graded as “very difficult” to “extremely difficult,” requiring a college graduate to professional-level reading ability. Overall, DeepSeek-V3 demonstrated the best readability, with a mean FRE score of 32.59, substantially higher than that of ChatGPT-3.5 (21.67) and Gemini-2.5 (18.70). Concurrently, its GFI (14.56), FKGL (11.58), and SMOG (10.23) scores were significantly lower, indicating a more accessible text structure and lexical choice. Conversely, Gemini-2.5 exhibited the poorest readability, with a mean FRE of 18.70 and corresponding GFI, FKGL, and SMOG scores of 18.23, 19.14, and 15.86, which equate to a “college graduate” to “professional” reading level. This suggests its responses often contained highly specialized jargon, protracted sentences, and complex concepts, rendering them largely inaccessible to the average reader. ChatGPT-3.5’s performance was intermediate, with all average scores falling within the “very difficult” to “professional” range, indicating that its texts also posed considerable challenges for non-specialists.

A comparative analysis of readability across different medical question categories revealed the most pronounced inter-model differences in responses to diagnostic questions. Notably, Gemini-2.5 consistently yielded the least readable texts, with a mean FRE score of 0.40, alongside markedly high mean FKGL and SMOG scores of 24.14 and 19.64, respectively. These scores correspond to a reading difficulty level comparable to that of specialized academic literature. This pattern suggests that when addressing highly technical topics, Gemini-2.5 tends to generate responses characterized by protracted sentences and dense jargon, posing significant challenges for patients seeking a foundational understanding. For instance, in its response to Q4, Gemini-2.5 produced text with exceptionally high FKGL (28.34) and SMOG (22.05) scores, indicating a minimal effort to simplify complex medical information. In contrast, DeepSeek-V3 demonstrated significantly superior readability across all metrics (mean FRE: 31.40; FKGL: 11.59; SMOG: 8.96), highlighting its enhanced capability to simplify diagnostic information. Nevertheless, its texts still required a reading level exceeding that of a high school graduate.

For long-term health risk questions, the readability of all models declined. Significant differences in the SMOG index were observed among the three models, with Gemini-2.5 maintaining extremely poor scores: a mean FRE of 3.50, alongside high mean GFI, FKGL, and SMOG scores of 20.08, 22.28, and 18.06, respectively. This indicates that Gemini-2.5 persisted in using an overly specialized writing style when explaining long-term risks. The scores for DeepSeek-V3 and ChatGPT-3.5 also decreased, suggesting that AI chatbots may generate more challenging text when explaining such complex and uncertain concepts. However, as these topics are non-acute and less terminology-intensive, they can sometimes allow for simpler explanations. For instance, DeepSeek-V3 achieved better readability scores on Q6 (FRE: 30.00, FKGL: 12.83) than Gemini-2.5 (FRE: 14.00, FKGL: 18.52). These contrasting trends underscore the high reading level demand posed by such content, highlighting the inconsistent readability performance of these models.

For treatment-related questions, the inherent length and complexity of therapeutic regimens resulted in generally low readability across all AI models. A key finding emerged, however, as DeepSeek-V3 achieved its highest mean FRE score (36.44) in this category, which was also significantly higher than that of both ChatGPT-3.5 and Gemini-2.5. Concurrently, its mean FKGL (10.96) and SMOG (9.92) scores were significantly lower than those of the other models. This finding is particularly critical because treatment plans often involve specific instructions whose clarity directly impacts patient safety and adherence. DeepSeek-V3’s superior performance may be attributed to its ability to decompose complex processes into shorter, more digestible sentences, potentially informed by explanations of common procedures or medications within its training data. Despite this relative improvement, its readability remained at a “difficult” level. His indicates that DeepSeek-V3 possesses a superior capacity for readability control when generating treatment-related content, enabling more effective simplification of inherently complex information.

## 4 Discussion

In this study, we systematically assessed the accuracy, quality, and readability of responses generated by ChatGPT-3.5, DeepSeek-V3, and Gemini-2.5 on hypothyroidism-related questions, using clinical guidelines as the reference standard. This enabled a comprehensive evaluation of their capabilities in the assessment and management of adult hypothyroidism. These models were chosen because of their broad public recognition and frequent use in medical contexts, thereby providing strong representativeness for the study. Previous studies have begun to examine the reliability and validity of AI chatbot outputs in thyroid disease (14, 15), especially Comparisons with current guidelines (9, 16), which have confirmed that AI chatbots are capable of generating high-quality professional medical information. This study specifically evaluated the quality and accuracy of AI-generated hypothyroidism-related information and further assessed readability to determine whether these responses could simultaneously meet professional medical standards and remain accessible to patients, thereby balancing authority with comprehensibility.

To optimize response quality, we engineered structured prompts that explicitly instructed each chatbot to generate responses commensurate with the expertise of a thyroid specialist, mandated grounding in evidence-based medicine, and required suitability for patient education. Notably, all three AI models demonstrated strong adherence to instructions, producing detailed responses that consistently referenced evidence-based sources. For instance, when asked about the use of L-T3 in hypothyroidism management, ChatGPT-3.5 explicitly endorsed the guideline’s Grade A recommendation of levothyroxine (L-T4) as the primary replacement therapy. It also noted that L-T3 monotherapy was ineffective and generally not recommended, consistent with a Grade F recommendation (strongly discouraged, with compelling evidence of lack of benefit or potential harm). The response further discussed recommendations regarding monotherapy with L-T3 or L-T4 as well as combination therapy, citing multiple evidence-based studies.

Previous studies have indicated that AI chatbots may not proactively clarify controversial aspects or well-established evidence in medical information (17), which imposes certain limitations on the professional depth of chatbot responses. In this study, the models were able to identify points of controversy and provide recommendations aligned with guideline grading. This suggests that through iterative training, AI chatbots may have developed a preliminary capacity for understanding, processing, balancing, and critically evaluating medical information (18). However, such capabilities remain limited. Our analysis was conducted using advanced, iteratively trained models and employed highly structured prompts. This raises the important consideration that users must be able to construct clear and specific instructions to elicit high-quality and accurate responses from AI chatbots. Accuracy and quality assessments revealed that all models achieved accuracy scores above 4.5 across categories, with no low ratings, demonstrating excellent reliability. Meanwhile, low-quality feedback was minimal. Both DeepSeek-V3 and Gemini-2.5 received no low-quality ratings, with overall high-quality response rates exceeding 94%, indicating an exceptionally high standard of answer quality. In summary, under appropriate prompting strategies, AI chatbots are capable of generating accurate, guideline-consistent, and high-quality content, thereby demonstrating substantial professional authority.

A potential issue identified in this study is that, although AI recommendations for hypothyroidism were generally accurate, the authenticity of cited evidence was questionable. For example, references were often vaguely described as “a study” without providing specific titles, retrievable identifiers, or source links. This phenomenon, known as “AI hallucination” (19), represents one of the most criticized shortcomings of AI, wherein fabricated or incorrect scientific references are provided. Frosolini et al. reported that only 16.66% of 120 references generated by ChatGPT were entirely accurate, with similar issues noted in other investigations (20, 21). It is important to emphasize that the frequency of AI hallucinations may not be high. In this study, the specific recommendations generated by AI chatbots for hypothyroidism were found to be of excellent accuracy and quality overall.

While the assessments of accuracy and quality were based on subjective evaluation scales, they were validated through inter-rater reliability (IRR) analysis, demonstrating moderate to strong consistency among raters. Furthermore, we acknowledge the value of computational linguistics metrics such as BERTScore, ROUGE, or BLEU in measuring textual semantic similarity. However, after careful consideration, we deliberately opted for expert manual evaluation based on clinical guidelines in this study. Semantic similarity metrics (e.g., BERTScore) inherently measure the linguistic proximity of words and sentences between AI-generated responses and reference guideline texts. Nevertheless, mere similarity is insufficient when evaluating clinical authority and medical accuracy, necessitating expert assessment. Expert reviewers not only verify factual correctness but, more importantly, evaluate the appropriateness of clinical context application and accurate adherence to evidence grading—for instance, whether models correctly distinguish between Grade A strong recommendations and Grade F against recommendations in guidelines. Pure semantic similarity algorithms cannot capture these nuances in clinical decision-making and evidence weighting. Secondly, only trained thyroid specialists can effectively identify AI-generated reference “hallucinations” (i.e., false or inaccurate citations) and assess the clinical correctness of medical facts beyond mere linguistic coherence. Therefore, to ensure the authoritative adherence of this study, we selected IRR-validated subjective evaluations conducted by two thyroid specialists for both accuracy and quality assessments.

It must be acknowledged that assessing semantic drift represents an ideal methodology for evaluating the trade-off between information fidelity and readability during medical text simplification. Information fidelity is paramount in healthcare communication. Implifying expert-level text (e.g., responses generated by specialist-caliber prompts) into patient-comprehensible versions inevitably involves a reduction in information granularity or complexity. BERTScore, an advanced NLP evaluation metric, calculates semantic similarity between candidate and reference texts by leveraging the contextual embeddings from BERT models and their variants. This semantics-based mechanism surpasses traditional lexical-overlap metrics (e.g., ROUGE) by capturing semantic equivalence at the sentence and phrase levels, making it particularly suitable for evaluating paraphrased yet meaning-preserving simplifications of medical information. The work of Rasool et al. (22), which evaluated models like nBERT in mental healthcare, further underscores the emerging trend of employing advanced BERT-based models for assessment in complex health contexts.

The findings of this study provide an instructive contrast to prior research evaluating AI chatbots in acute or highly specialized medical contexts. For instance, previous studies have demonstrated the efficacy of AI in tackling the USMLE (5), ophthalmology Q&A (6), and ENT surgery (7), primarily focusing on its potential as a clinical decision-support tool or knowledge base. In contrast, the unique contribution of this work lies in shifting the focus from technical proficiency to AI’s performance in the broader and more challenging application scenario of patient education. By conducting a systematic evaluation of accuracy, quality, and readability within a unified framework focused on hypothyroidism, this study reveals a critical practical challenge: a significant gap persists between the technical perfection of AI-generated information and its translation into patient-comprehensible language. This insight not only informs the future development of AI in healthcare but also underscores the necessity of prioritizing readability as a co-equal metric to accuracy in developing patient-facing AI applications.

Although AI chatbot responses were accurate and of high quality, reflecting professional authority, their readability was not equally strong, potentially limiting patient-friendliness. In this study, chatbot-generated content was generally rated as “very difficult” or “extremely difficult” to read, often requiring a college graduate or professional-level background. All models exhibited particularly low readability for diagnostic-related content. For instance, Gemini-2.5 scored an FRE of 0.00 across four diagnostic questions, indicating that its responses were virtually incomprehensible to non-specialists. This consistently extreme difficulty suggests that current AI chatbots may struggle to maintain accuracy while simultaneously translating complex diagnostic criteria and terminology into lay language (23). This pattern may stem from training datasets predominantly composed of highly specialized sources, such as medical journals, clinical guidelines, and textbooks. Medical texts inherently contain specialized terminology (e.g., “levothyroxine,” “thyroid-stimulating hormone”). These polysyllabic terms and complex concepts naturally elevate scores on computational indices such as SMOG and GFI, resulting in generally low readability ratings. The findings of this study—that all model outputs were classified as “very difficult” to “extremely difficult” to read, requiring a “college graduate” to “professional” education level—precisely quantify this inherent complexity. While this can yield technically precise outputs, they are often unsuitable for laypersons. Such a mode of generation, while potentially valuable for professional use, risks causing misunderstanding, anxiety, and non-adherence if directly presented to patients. It should be noted that prompts were calibrated to a specialist-physician level to ensure a baseline of accuracy, which inherently justifies the elevated reading level of the responses.

Furthermore, to provide a more nuanced interpretation, we analyzed variations across different question categories and observed that readability was influenced not only by lexical complexity but also by information structure. The medical topic category itself likely influences AI readability. Contrary to the assumption that diagnostic information is the most technical and treatment plans are more procedural, our data revealed a more nuanced picture. However, our data revealed that in certain instances, AI-generated responses to treatment-related questions (Q10–Q18) demonstrated higher readability than those addressing diagnostic inquiries (Q1–Q5). Notably, DeepSeek-V3 achieved its highest mean FRE score (36.44) specifically in treatment-related responses. Conversely, readability measures decreased again when models addressed long-term risk questions (Q6–Q9). This variation suggests that AI may better organize and simplify language for well-structured, sequential information like treatment steps, thereby enhancing readability. Conversely, when addressing terminology-intensive topics such as diagnosis or abstract concepts like long-term health risks, models tend to revert to a more rigid and complex default generation mode, consequently reducing readability. For instance, Gemini-2.5’s responses to diagnostic questions yielded a near-zero mean FRE score while requiring an exceptionally high reading level (FKGL 28.34), demonstrating how technical complexity constrains accessibility in these domains.

The readability levels of chatbot responses in this study exceeded the thresholds recommended by the U.S. National Institutes of Health. Continued output at such high difficulty would render the information inaccessible to substantial populations, such as those with lower literacy, non-native speakers, or individuals with cognitive impairment. Difficult-to-understand online health information may foster the spread of misinformation, potentially jeopardizing individual health (24). This phenomenon is not unique. Momenaei (27) observed that ChatGPT outputs on retinal surgery were difficult for laypersons to read, requiring at least a college education. Similarly, Mine Büker et al. (28) reported that all chatbot responses regarding root canal retreatment exceeded recommended readability thresholds, being suitable only for readers at grade 10 or higher. These findings indicate that, although AI chatbots hold significant potential in the evaluation and management of adult hypothyroidism, they offer valuable support to both information-seeking patients and healthcare professionals seeking rapid, reliable insights. However, the inherent complexity of medical terminology, disease mechanisms, and treatment protocols, coupled with variations in health literacy and educational background, may create substantial comprehension barriers. Therefore, it’s essential to ensure that AI chatbots provide not only accurate information but also content that is easy to understand.

Previous studies have attempted to improve the readability of AI-generated outputs. Xu et al. (29) improved ChatGPT’s ability to support patient understanding and management of thyroid cancer by adjusting prompt strategies to generate responses more aligned with patient needs. Jacob et al. (30) enhanced readability by re-entering ChatGPT-generated content into the model to produce simplified summaries understandable at a sixth-grade level. James et al. (31) used the prompt “explain the radiology report to the patient in plain second-person language” with AI-LLMs, achieving significantly improved FKGL and FRE scores. Multiple studies have demonstrated the potential of AI chatbots as tools for patient education. Qais et al. (32) confirmed that large language models could generate highly readable, accurate, and high-quality patient education material on dry eye disease. Zhang (33) demonstrated, through patient evaluation, that large language models could serve as effective educational tools for individuals with inflammatory bowel disease. Collectively, these studies indicate that strategies such as prompt optimization can effectively enhance the readability of chatbot-generated content, making it more patient-friendly. This suggests that AI language models might benefit from offering separate user interfaces tailored to clinicians and patients, thereby balancing professional authority with accessibility.

This study has several limitations. First, only English responses were analyzed; incorporating non-English queries could broaden the scope of evaluation and enhance the generalizability of findings. Second, only three chatbot models were evaluated. Given the rapid evolution of the field and the continual emergence of new models, future research should include a wider range of systems to improve the robustness of conclusions. Finally, the dynamic processes inherent in real-world use—such as multi-turn dialogue, seeking clarification, and request rephrasing—are critical for fully assessing the potential of AI in patient education. A fixed set of predefined questions may not capture model performance in response to ambiguous or unstructured queries, and single-turn interactions fail to assess coherence and memory across complex, multifaceted discussions. Consequently, evaluations conducted under these constrained conditions may yield overly optimistic estimates of chatbot capabilities. Meanwhile, the lack of assessment for potential demographic or cultural biases represents a significant limitation of this study. We emphasize that future research must employ multi-turn dialogue designs and patient-generated free-form queries to simulate more authentic interaction scenarios, in order to fully capture fairness or bias issues that may arise when providing personalized treatment recommendations, lifestyle modifications, or dietary guidance.

## 5 Conclusion

This study systematically evaluated the performance of ChatGPT-3.5, DeepSeek-V3, and Gemini-2.5 in responding to hypothyroidism-related questions in adults. All three models were capable of generating highly accurate and high-quality medical information, with responses largely aligned with clinical guidelines, highlighting the strong potential of AI in supporting medical knowledge dissemination. However, the readability of outputs across all models was generally low, which may limit their effectiveness in patient education. Future research should focus on strategies such as prompt engineering and multi-turn dialogue optimization to improve readability and patient-friendliness while maintaining professional accuracy, thereby enabling broader application of AI in health education.

## Data Availability

The raw data supporting the conclusions of this article will be made available by the authors, without undue reservation.

## Footnotes

1
https://chat.openai.com/

2
https://deepseek.com/

3
https://gemini.google.com/app

4
http://readabilityformulas.com/
